# Integrative care: acupuncture based neuromodulation therapy for diabetes and heart failure

**DOI:** 10.3389/fnins.2024.1332957

**Published:** 2024-01-17

**Authors:** Wei Zhou, Andy Lee, Aren Zhou, Dawn Lombardo

**Affiliations:** ^1^Division of Cardiology, University of California, Irvine, Orange, CA, United States; ^2^Irvine Valley College, Irvine, CA, United States

**Keywords:** neuromodulation, acupuncture, brain stem, neurotransmitters, central nervous system, heart failure, diabetes

## Abstract

The relationship between heart failure and diabetes is intricate and bidirectional. Individuals with diabetes face an elevated risk of developing heart failure due to factors like insulin resistance, chronic inflammation, and metabolic irregularities. Elevated blood sugar levels can harm blood vessels and nerves, culminating in the buildup of fatty deposits in arteries, atherosclerosis, and hypertension, which significantly contribute to heart failure. Furthermore, diabetes can adversely impact the structure and function of the heart muscle, impairing its pumping capacity. Conversely, heart failure can also contribute to the onset of diabetes by disrupting the body’s metabolic processes and amplifying insulin resistance. The complex interaction between these conditions mandates a comprehensive approach to managing individuals with both diabetes and heart failure, underscoring the importance of addressing both aspects for enhanced patient outcomes. Although existing pharmacological treatments are limited and frequently associated with undesirable side effects, acupuncture has established itself as a traditional practice with a legacy. It remains a supplementary option for treating cardiovascular diseases. Heart failure and diabetes are both heavily associated with chronic upregulation of the sympathetic nervous system, which has been identified as a pivotal factor in the progression of disease. Mechanistic interplays such as the attenuation of central nitric oxide signaling may interfere with the production or availability of nitric oxide in key areas of the central nervous system, including the brainstem and hypothalamus. This review will delve into the current understanding of acupuncture on the autonomic nervous system and offer insights into its potential role in the future treatment landscape for diabetes and heart failure.

## Introduction

Cardiovascular diseases rank as the primary cause of death worldwide. The major risk factors associated with the development of cardiovascular conditions, including left ventricular hypertrophy, coronary artery disease, and heart failure, are diabetes and hypertension. The prevalence of diabetes rises with age, with a lifetime risk approaching 32.8% for males and 38.5% for females (Narayan et al., 2003; Haji Mohd Yasin et al., 2014). The relationship between heart failure and diabetes is intricate and bidirectional. Diabetic patients have an increased risk of developing heart failure due to factors such as insulin resistance, chronic inflammation, and metabolic abnormalities. Elevated blood sugar levels can inflict harm on blood vessels and nerves, leading to the buildup of fatty deposits in arteries, atherosclerosis, and hypertension – all significant contributors to heart failure. Furthermore, diabetes can adversely affect the heart’s structure and function thereby impairing its pumping ability. Conversely, heart failure may play a role in diabetes risk by disrupting the body’s metabolic processes and increasing insulin resistance. The intricate interplay between these two conditions underscores the need for a comprehensive approach to managing individuals with both diabetes and heart failure, emphasizing the importance of addressing both aspects to enhance overall patient outcomes. While treatment strategies (Berger and Muhlhauser, 1999; Bansal et al., 2007; Chen et al., 2019) have been devised to target hypertension, hyperglycemia, and dyslipidemia, in addition to lifestyle modifications, for this disease, a universal treatment has not yet been identified as conventional therapies often entail various adverse side effects.

Acupuncture is gaining increasing acceptance as a complementary and alternative therapy in the American healthcare system. In East Asia, forms of acupuncture including manual acupuncture and electroacupuncture (EA) have been utilized to address various cardiovascular diseases including coronary heart disease, hypertension, diabetes, and heart failure (Middlekauff et al., 2004; Peplow and Baxter, 2012; Zhou and Longhurst, 2012; Lee et al., 2016). Nevertheless, numerous Western physicians hesitate to endorse acupuncture due to the ongoing controversies surrounding its efficacy in treating diabetes and heart failure. Furthermore, physiological mechanisms underpinning these effects remain largely elusive to Western medicine practitioners. This brief review delves into the existing comprehension of the physiological effects of acupuncture and provides perspectives on the potential future contributions of neuromodulation therapies, including EA, in managing diabetes and heart failure.

### Background knowledge of acupuncture and electroacupuncture and effects related to diabetes and heart failure

Acupuncture, an ancient practice originating from traditional Chinese medicine and spanning over 3,000 years, involves the insertion of needles at specific points along meridians within the human body to address imbalances in ‘Qi,’ representing the flow of energy. Initially crafted from bronze, silver, and gold, acupuncture needles have transitioned to contemporary use, predominantly made of stainless steel and available in various lengths (ranging from 0.5 to 100 mm) and diameters (ranging from 0.12 to 0.30 mm). The depth of needle insertion varies, encompassing shallow (1–2 mm) to deep needling (50–60 mm), with diverse methodologies eliciting distinct responses. As an illustration, when employing EA, stimulation of acupoints located above deep somatic nerves may induce inhibitory effects on cardiovascular reflex responses. In contrast, acupoints situated over superficial nerves do not exhibit the same effects, suggesting the presence of point specificity in acupuncture treatment (Tjen-A-Looi et al., 2004).

A literature review by Peplow and Baxter focused on EA’s role in blood glucose control in diabetes (Peplow and Baxter, 2012). Among the limited human studies identified, only two employed EA, demonstrating efficacy in improving glycemic in obese patients as well as healthy subjects (Szczudlik and Lypka, 1984; Cabioglu and Ergene, 2006). In mammalian models, EA at 15 Hz for 30 min at Zusanli and Zhongwan’s acupoints manifested a blood glucose-lowering effect in fasting diabetic rats (Amundsen et al., 2006; Chang et al., 2006; Ishizaki et al., 2009) and in a fasting normal rat model (Tseng et al., 2005). Remarkably, EA at these acupoints in hyperglycemic rats demonstrated normalization in glucose levels and elevations in serotonin and endogenous opioid levels (Lin et al., 2004; Chang et al., 2006).

### Role of autonomic nervous system in development of diabetes and heart failure

The autonomic nervous system (ANS) consists of the parasympathetic and sympathetic systems, playing a crucial role in regulating glucose metabolism and affecting both insulin secretion and absorption. Numerous research studies have consistently shown a reciprocal and causative link between ANS dysfunction and diabetes. Elevated sympathetic activity may have multiple underlying causes, including a genetic polymorphism within the beta-2 and beta-3 adrenergic receptor genes, which is directly associated with diabetes (Masuo et al., 2010). Sympathetic overactivity stimulates the renin-angiotensin-aldosterone (RAA) system, facilitating sodium reabsorption and elevating heart rate, stroke volume, and peripheral vascular resistance. Consequently, this cascade induces hypertension and heightens cardiovascular risk (Perin et al., 2001). Hyperinsulinemia in type 2 diabetes is also associated with sympathetic nervous system activation which results in increased systemic vascular resistance and renal sodium reabsorption, thereby increasing the risk of developing hypertension (Perin et al., 2001). Additionally, autonomic remodeling in cardiomyocytes has been observed in diabetic rat models and is characterized by increased heterogeneity in atrial effective refractory periods and alterations in sympathetic nerve histology, as revealed by tyrosine hydroxylase staining. These changes render diabetic rats more susceptible to atrial fibrillation (Thackeray et al., 2012).

Within the diabetic heart, sympathetic overactivity redirects energy metabolism toward fatty acid utilization rather than glucose. This shift may lead to insulin resistance, intracellular lipid metabolite accumulation, increased oxidative stress, and cardiac fibrosis, all of which may culminate in reduced contractile capacity and disruption of natural cardiac systolic and diastolic function (Zwseykm, 2013). The sympathetic nervous system (SNS) may be activated as an adaptation for impaired systolic function (Thackeray et al., 2012). Elevated serum levels of epinephrine and norepinephrine, indicative of sympathetic activation, are often detected in type 1 diabetic patients at high risk for diabetic kidney disease (Perin et al., 2001). In type 2 diabetes patients, sympathetic activation may be associated with insulin resistance, stimulating both alpha- and beta-adrenergic receptors within skeletal muscle, leading to reduced vasodilation and contributing to systemic hypertension (Esler, 1995; Esler et al., 2001; Carnethon et al., 2003; Carnethon and Craft, 2008). The increased prevalence of systemic hypertension in type 2 diabetes patients, as opposed to type I, may be further explained by the direct association between hyperglycemia, caloric intake, and body mass index (Troisi et al., 1991).

On the other hand, the parasympathetic system promotes insulin secretion by pancreatic beta cells, while the sympathetic branch inhibits insulin release (Carnethon et al., 2003). When vagal tone is impaired or reduced, the balance between sympathetic and parasympathetic activity may be disrupted. As a result, a decrease in vagal tone may promote sodium reabsorption as well as increased stroke volume, higher heart rate, and elevated systemic vascular resistance. All of these factors may serve as risk factors in hypertension and cardiovascular issues, both of which contribute to risk of diabetes. Studies have suggested that younger adult patients with impaired vagal tone often exhibit hyperinsulinemia (Carnethon et al., 2003). Furthermore, chronic hyperglycemia may promote a progressive degeneration of autonomic nerves, mirroring the development of peripheral neuropathy. This degeneration is primarily evident in the vagal nerve, which is the longest autonomic neural fiber and is often associated in patients with cardiac autonomic neuropathy (CAN) (Carnethon et al., 2003). Diabetic patients may initially experience parasympathetic denervation with increased sympathetic tone, leading to nocturnal hypertension, and progress to sympathetic denervation, leading to orthostatic hypotension. Examining heart rate variability can offer valuable insights into cardiac autonomic modulation, serving as a potent tool for the prevention, diagnosis, and treatment of cardiovascular diseases, including CAN. The advancement of CAN may potentially result in both arrhythmias and sudden death (Vinik and Ziegler, 2007).

Obesity is linked to problems in the autonomic nervous system, making the body less responsive to insulin and affecting how blood vessels function. This can lead to issues like high blood pressure, increased insulin levels, oxidative stress, and the development of scar tissue in the heart muscle. These mechanisms are associated with diabetes (Parati and Esler, 2012). It is clear that the diabetic condition and sympathetic overactivity mutually exacerbate each other. The sympathetic nervous system has a pivotal role in the progression of diabetes as well as heart failure. An imbalance between sympathetic and vagal activity has been demonstrated to worsen the prognosis and symptoms of heart failure. To address this, a therapy called baroreflex activation, which involves electrically stimulating the baroreflex, has been developed. Coats et al. conducted a meta-analysis revealing that baroreflex activation therapy enhances exercise capacity, reduces NYHA class, lowers NT-proBNP levels, and improves overall quality of life in heart failure patients who are also receiving guideline-directed medical therapy. This improvement is achieved through sympathoinhibition (Coats et al., 2022).

In the last two decades, our research has concentrated on investigating the impact of acupuncture on the central neuroregulation of sympathoexcitatory reflexes. This involves exploring different brain regions, including the rostral ventral lateral medulla (rVLM), hypothalamic arcuate nucleus (ARC), midbrain ventrolateral periaqueductal gray (vlPAG) nuclei, medullary nucleus raphé pallidus (NRP), as well as the dorsal horn and intermediolateral column of the spinal cord.

### Autonomic effects of electroacupuncture

The rVLM is a region located in the brainstem, specifically within the medulla oblongata. Its neuroanatomical location is situated rostral and ventral in relation to the medulla (Ally, 1998). Afferent connections to the rVLM involve inputs from various sources, including higher brain centers such as the hypothalamus and cerebral cortex. Additionally, sensory information from peripheral receptors, like baroreceptors and chemoreceptors, contributes to the afferent signaling to the rVLM (Ally, 1998). These afferent connections provide the rVLM with information about blood pressure, oxygen levels, and other relevant physiological parameters. Efferent connections from the rVLM primarily involve the sympathetic nervous system. The rVLM contains presympathetic neurons that project to the intermediolateral cell column of the spinal cord, which is responsible for regulating sympathetic outflow to different target organs. These efferent connections play a crucial role in controlling both vasomotor (blood vessel tone) and non-vasomotor (non-blood vessel related) sympathetic activities.

The rVLM presympathetic neurons are particularly involved in the regulation of cardiovascular functions (Dampney et al., 2003). They modulate vasomotor sympathetic outflow, influencing blood pressure and blood vessel tone. Additionally, rVLM activity is implicated in the control of non-vasomotor sympathetic activities, such as the regulation of respiratory and metabolic functions.

In summary, the rVLM is a key region in the brainstem that plays a vital role in the autonomic control of cardiovascular and other physiological functions. It receives input from various sources, integrates this information, and sends output to the sympathetic nervous system, exerting control over both vasomotor and non-vasomotor sympathetic activities. The rVLM regulates sympathetic outflow and blood pressure (Guyenet, 1990), and the significant reduction in sympathetic outflow is achieved by inhibiting neuronal activity in the rVLM (Guertzenstein and Silver, 1974).

The renin-angiotensin system (RAS) plays a pivotal role in regulating the SNS within the brain (Jankowska et al., 2006). In experimental chronic systolic heart failure, there is heightened sympathetic outflow accompanied by the activation of the brain RAS system (Wang and Zucker, 1996). Notably, angiotensin II type 1 (AT1) receptors are prominently expressed in key regions of the hypothalamus and medulla, which exert control over sympathetic outflow (Zucker et al., 2009). Furthermore, aldosterone has the potential to elevate AT1 receptor levels in the paraventricular nucleus (PVN) of the hypothalamus. The mechanism through which RAS induces sympathetic excitation involves brain reactive oxidative stress. It is firmly established that the activation of AT1 receptors can instigate oxidative stress in the rVLM. In animal models of chronic HF, the microinjection of angiotensin II into the rVLM results in sympathoexcitation, while the microinjection of AT1 receptor blockers into the rVLM induces sympathoinhibition.

Earlier investigations have indicated that low-frequency EA and manual acupuncture have the potential to suppress the reflex response and premotor sympathetic neural firing in the rVLM (Zhou et al., 2005). This modulation of EA is nullified by the administration of naloxone (a non-specific opioid receptor antagonist) and gabazine (a type A gamma-aminobutyric acid (GABA) receptor inhibitor) to the rVLM (Tjen-A-Looi et al., 2003). The rVLM processes somatic input during acupuncture stimulation, and electrophysiological studies have demonstrated that certain acupoints (P 5–6 and LI 4–11) over deep median and radial nerves provide increased afferent input to cardiovascular premotor sympathetic neurons in the rVLM compared to cardiovascular inactive acupoints over superficial afferent nerves (LI 6–7, G 37–39) (Tjen-A-Looi et al., 2004). This observation explains why acupuncture over the deep median and radial nerves is effective in attenuating BP and sympathetic outflow.

Previous studies have documented the mitigating effects of EA on sympathoexcitatory reflexes mediated by endogenous opioids, GABA, nociceptin, and serotonin (5-hydroxytryptamine, 5-HT) in the rVLM (Huangfu and Li, 1988a,b; Crisostomo et al., 2005; Li et al., 2006). Our research has revealed that EA’s inhibition of sympathoexcitatory reflex responses in cats is attributed to activation of μ- and δ-opioid receptors in the rVLM, suggesting the involvement of enkephalins, endorphins, and possibly endomorphin in EA’s modulation of cardiovascular responses. Immunohistochemical staining has identified enkephalinergic neurons in the rVLM and endorphinergic neurons in the ARC, which project directly to the rVLM. Both neurotransmitter systems are activated by EA (Guo and Longhurst, 2007). EA inhibits the sympathetic reflex response by opioid-mediated inhibition of glutamate in the rVLM (Zhou et al., 2007). Electrophysiological studies have demonstrated the existence of reciprocal excitatory glutamatergic projections between the ARC and vlPAG, potentially contributing to EA’s inhibition of sympathetic nerve activity. This reciprocal projection may also involve a cholinergic component in the ARC, but not in the vlPAG (Dampney et al., 2002, 2003).

Moreover, EA, achieves a reduction in the vlPAG release of GABA but not glutamate through presynaptic endocannabinoid CB1 receptor stimulation. This decrease in GABA disinhibits vlPAG neurons, increasing their activity, and subsequentlyinhibiting rVLM cardiovascular sympathetic neurons and associated sympathoexcitatory reflex responses during EA. Hence, the cardiovascular modulation by EA involves a range of neurotransmitter systems, encompassing both excitatory and inhibitory neurotransmitters, with their importance varying across different brain regions.

Extensive research has delved into the interaction among the hypothalamic ARC, the vlPAG and rVLM in the context of EA and cardiovascular sympathoexcitatory responses (Dampney et al., 2002). Microinjections of excitatory amino acids into the ARC can enhance vlPAG neuronal responses. Conversely, kainic acid (KA) microinjection into the ARC induces a reversible depolarization blockade, transiently deactivating arcuate neurons and reducing vlPAG responses to splanchnic nerve (SN) stimulation. EA amplifies SN-evoked discharge of vlPAG neurons, a response blocked by KA microinjection into the ARC. These findings underscore the crucial role of excitatory projections from the ARC to the vlPAG in the inhibitory impact of EA on the pressor reflex induced by SN and gallbladder afferent stimulation (Li et al., 2009).

The vlPAG delivers inhibitory input to premotor sympathetic neurons in rVLM, ultimately diminishing sympathetic outflow and reflex increases in blood pressure. Direct axonal projections from the vlPAG to the medulla exist. Recent research indicates that the nucleus raphé pallidus (NRP), positioned more ventrally than the nucleus raphé obscurus (NRO), exhibits increased activation during median nerve stimulation with electroacupuncture (EA) at specific acupoints (Tjen-A-Looi et al., 2004, 2006). Temporary chemical blockade of the NRP reverses the activation of neurons in the rVLM during vlPAG stimulation, as well as EA modulation of visceral excitatory reflexes (Tjen-A-Looi et al., 2004, 2006). Serotonin projections from the raphé, acting on 5-HT1A receptors in the rVLM, complete the vlPAG-NRP-rVLM circuit, regulating cardiovascular activity. Thus, an indirect connection from the vlPAG to the rVLM, involving a serotonergic link between the NRP and the rVLM, plays a crucial role in the long-loop modulation of cardiovascular sympathetic outflow during reflex visceral stimulation.

Neurons in vlPAG receive convergent input from somatic nerves stimulated during EA and from the ARC. The blockade of the caudal vlPAG results in the reversal of arcuate-evoked rVLM responses, as indicated by retrograde tracer studies showing that arcuate perikarya are labeled with retrograde tracers microinjected into the rVLM. Many neurons originating from the arcuate and projecting to the rVLM are activated by EA stimulation, often containing opioid peptides, particularly β-endorphin. Hence, the vlPAG, particularly the caudal vlPAG, appears crucial for inhibiting rVLM neuronal activation by the ARC and subsequent EA-related cardiovascular activation. Additionally, direct projections from the ARC to the rVLM likely serve as a significant source of β-endorphin, given the presence of this opioid peptide in this projection. The spinal cord plays a pivotal role in processing somatic and visceral reflexes, as well as receiving outputs from the central nervous system that impact effector organs participating in cardiovascular reflex regulation (Longhurst, 2003). Additionally, EA exerts cardioprotection against myocardial ischemia by triggering the release of endogenous opioids in the PVN (Wang et al., 2005).

Notably, the dorsal horn of the spinal cord serves as a major center for EA-induced analgesia, with EA leading to an increase in Fos immunoreactive neurons in the superficial laminae of the dorsal horn (Lee and Beitz, 1992, 1993). Opioid and nociceptin-like immunoreactivity are identified in the spinal sympathetic nuclei, where opioids and nociceptin can modulate sympathetic outflow. Descending pathways from the brainstem to the dorsal horn of the spinal cord may also exert influence on the segmental processing of somatic inputs during EA (Han et al., 1982; Han and Xie, 1984; Zhou et al., 2009). Interneurons in the dorsal horn exhibit responses to somatic stimulation, demonstrating either excitatory or inhibitory characteristics based on dermatome stimulation. These interneurons play a vital role in the spinal cord circuitry associated with autonomic control. However, additional research is necessary to gain a comprehensive understanding of the spinal circuits governing cardiovascular visceral reflex responses during EA. Previous studies have shown that EA modulates body physiology at distant sites via somatosensory autonomic reflexes (Liu et al., 2020, 2021). For instance, Low-intensity EA applied to hindlimb regions stimulates the vagal-adrenal axis, leading to the production of anti-inflammatory effects reliant on NPY(+) adrenal chromaffin cells. In contrast, high-intensity ES administered to the abdomen triggers the activation of NPY(+) splenic noradrenergic neurons through the spinal-sympathetic axis. These neurons participate in incoherent feedforward regulatory loops via the activation of distinct adrenergic receptors (ARs). The resulting EA-induced activation can elicit either anti- or pro-inflammatory effects, influenced by disease-state-dependent changes in AR profiles. The identification of somatotopic organization and intensity dependence in driving specific autonomic pathways may serve as a guide for optimizing stimulation parameters. This optimization could enhance both the efficacy and safety of utilizing acupuncture as a therapeutic modality.

### Summary

**Figure 1:**
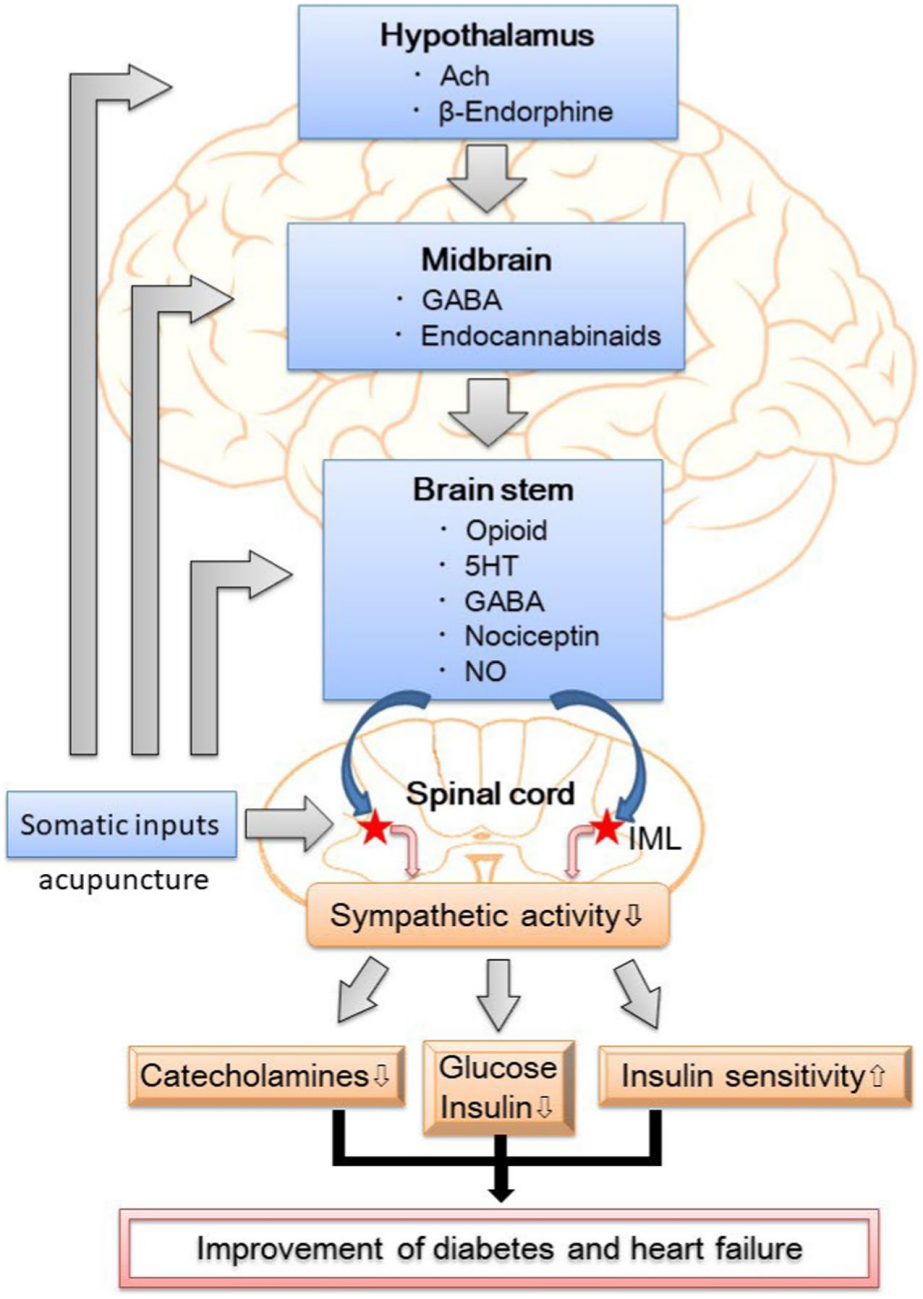
The putative neural mechanism of hypoglycemic and sympathoinhibitory responses to acupuncture. GABA, *γ*-aminobutyric acid; 5HT, 5- hydroxytryptamine or serotonin; NO, nitric oxide; IML, intermediolateral column of the spinal cord; DM, diabetes mellitus.

EA attenuates sympathetic outflow by affecting cardiovascular presympathetic neuronal activity in the rVLM probably through somatosensory autonomic reflex. Neurons located in the ARC of the hypothalamus, PVN, vlPAG in the midbrain, and NRP in the medulla may be activated by EA, leading to the inhibition of premotorsympathetic neurons in the rVLM. Various neurotransmitters, including acetylcholine, endogenous opioids, GABA, glutamate, nociceptin, NO, serotonin, and endocannabinoids, are involved in the sympathoinhibitory response mediated by EA (Figure 1). Since the ANS is a vital component of glucose metabolism, the modulation of sympathetic outflow may be the physiologic basis of hypoglycemic and sympathoinhibitory effects from acupuncture. Prior research has shown that EA decreases plasma glucose by increasing insulin production and improving insulin sensitivity as well as decreases sympathetic nerve activity through the secretion of endogenous β-endorphin and serotonin. These findings provide a strong connection between central neural regulation by acupuncture and the treatment of diabetes and heart failure.

### Prospects ahead: looking into the future

Traditional medical interventions for diabetes and heart failure are not without drawbacks, potentially leading to side effects. A growing body of evidence suggests that acupuncture, as a form of neuromodulation, could be an effective treatment for both conditions. EA has demonstrated the ability to reduce blood glucose levels, enhance insulin sensitivity, and decrease sympathetic nerve activity, all without associated side effects. We propose that incorporating lifestyle modifications and integrative approaches, including acupuncture, could be a reasonable initial therapy for diabetes and heart failure. Moreover, for individuals already receiving pharmacologic therapy for diabetes or heart failure, adopting lifestyle changes and exploring alternative treatments, particularly acupuncture, may contribute to improved glycemic control and allow for optimization in the dosages of standard pharmacologic agents. Nevertheless, additional research investigating the combination of acupuncture with drug therapies in the context of diabetes and heart failure is imperative.

## Author contributions

WZ: Writing – original draft, Conceptualization, Writing – review & editing. AL: Writing – review & editing, Conceptualization, Funding acquisition. AZ: Data curation, Software, Visualization, Writing – review & editing. DL: Conceptualization, Writing – review & editing.
